# Antimicrobial Cu-Doped TiO_2_ Coatings on the β Ti-30Nb-5Mo Alloy by Micro-Arc Oxidation

**DOI:** 10.3390/ma17010156

**Published:** 2023-12-27

**Authors:** Giovana Collombaro Cardoso, Katia Barbaro, Pedro Akira Bazaglia Kuroda, Angela De Bonis, Roberto Teghil, Ivan I. Krasnyuk, Luca Imperatori, Carlos Roberto Grandini, Julietta V. Rau

**Affiliations:** 1 Laboratório de Anelasticidade e Biomateriais, UNESP—Universidade Estadual Paulista, Bauru 17033-360, SP, Brazil; pedro.kuroda@unesp.br (P.A.B.K.); carlos.r.grandini@unesp.br (C.R.G.); 2 Istituto di Struttura della Materia, Consiglio Nazionale delle Ricerche (ISM-CNR), Via del Fosso del Cavaliere 100, 00133 Rome, Italy; luca.imperatori@cnr.it (L.I.); giulietta.rau@ism.cnr.it (J.V.R.); 3 Istituto Zooprofilattico Sperimentale Lazio e Toscana “M. Aleandri”, Via Appia Nuova 1411, 00178 Rome, Italy; katia.barbaro@izslt.it; 4 Dipartimento di Scienze, Università della Basilicata, Via dell’Ateneo Lucano 10, 85100 Potenza, Italy; angela.debonis@unibas.it (A.D.B.); roberto.teghil@unibas.it (R.T.); 5 Department of Analytical, Physical and Colloid Chemistry, Institute of Pharmacy, Sechenov First Moscow State Medical University, Trubetskaya 8, Build. 2, 119048 Moscow, Russia; krasnyuk_i_i_1@staff.sechenov.ru

**Keywords:** antimicrobial, antibacterial, micro-arc oxidation, copper, coating, surface modification, titanium alloy

## Abstract

Among the different surface modification techniques, micro-arc oxidation (MAO) is explored for its ability to enhance the surface properties of Ti alloys by creating a controlled and durable oxide layer. The incorporation of Cu ions during the MAO process introduces additional functionalities to the surface, offering improved corrosion resistance and antimicrobial activity. In this study, the β-metastable Ti-30Nb-5Mo alloy was oxidated through the MAO method to create a Cu-doped TiO_2_ coating. The quantity of Cu ions in the electrolyte was changed (1.5, 2.5, and 3.5 mMol) to develop coatings with different Cu concentrations. X-ray diffraction, X-ray photoelectron spectroscopy, scanning electron and atomic force microscopies, contact angle, and Vickers microhardness techniques were applied to characterize the deposited coatings. Cu incorporation increased the antimicrobial activity of the coatings, inhibiting the growth of *Staphylococcus aureus*, *Enterococcus faecalis*, *Pseudomonas aeruginosa* bacteria strains, and *Candida albicans* fungus by approximately 44%, 37%, 19%, and 41%, respectively. Meanwhile, the presence of Cu did not inhibit the growth of *Escherichia coli*. The hardness of all the deposited coatings was between 4 and 5 GPa. All the coatings were non-cytotoxic for adipose tissue-derived mesenchymal stem cells (AMSC), promoting approximately 90% of cell growth and not affecting the AMSC differentiation into the osteogenic lineage.

## 1. Introduction

Titanium (Ti) and its alloys are widely studied in the biomaterials field, mainly for producing dental and orthopedic implants. Ti is a transition metal that can form solid solutions with other elements with similar atomic sizes [1]. At temperatures up to 883 °C, it has a hexagonal closest-packed (hcp) crystal structure, known as the α phase. Above this temperature, a phase transition occurs, and it has a body-centered cubic (bcc) structure known as the β phase [2]. Depending on the alloying element used, the transition temperature (β-transus) can increase (α-stabilizing elements) or decrease (β-stabilizing elements) [3].

It is known that the best elastic modulus values are found in metastable β-type alloys, thus avoiding the stress-shielding effect caused by the mechanical incompatibility between the implant and the bone [4]. Therefore, using β-stabilizing alloy elements, such as Mo, Nb, and Ta, is preferable to develop Ti alloys with mechanical properties more suitable for use as implant biomaterials [5,6,7,8,9,10].

The Ti-30Nb-5Mo alloy, a newly developed β-metastable alloy [11,12], is more wear-resistant due to its higher hardness compared to the commercially pure Ti (CP-Ti), and it contains niobium (Nb), which can naturally form a protective layer of TiO_2_ and Nb_2_O_5_, thereby enhancing its corrosion resistance [13,14]. The alloy has an elastic modulus (69 GPa) closer to that of human bone, reducing the “stress shielding” effect [2].

However, even with the excellent properties that Ti alloys present, there is still a lack of bioactivity on their surface. In this way, the functionalization of the surface of the alloys is sought to be imparted by different methods [15]. One of the most straightforward and versatile one is the micro-arc oxidation (MAO) technique, also called plasma electrolytic oxidation (PEO) [16,17,18].

Surface treatment via the MAO technique allows the production of a thin porous layer with different properties, which protects the substrate (alloy) from corrosion and wear and reduces the release of potentially harmful ions from the alloy [19,20]. Furthermore, it also allows the incorporation of essential ions to promote cell adhesion and osseointegration and provides antimicrobial activity [18,21].

The elements most incorporated into coatings to obtain antimicrobial activity are silver (Ag), copper (Cu), and zinc (Zn) [22,23,24,25,26]. Among these elements, it has already been reported that materials incorporated with Cu also exhibit higher cytocompatibility [27]. Furthermore, the presence of Cu in biomaterials can improve angiogenic and osteogenic activities [28]. By interfering with DNA replication and causing disruption of the cell membrane of both Gram-positive and Gram-negative bacteria, Cu has excellent antibacterial properties [29,30,31]. In the literature, there are still no reports about the production of MAO coatings on the Ti-30Nb-5Mo alloy containing Cu as a bactericidal agent.

In the current study, TiO_2_ MAO coatings incorporating Mg are generated to enhance the biocompatibility of Ti alloys for orthopedic applications [32,33]. Magnesium (Mg) is a biocompatible metal that exhibits a natural affinity with biological tissues. This property reduces the probability of rejection or adverse reactions within the body [34].

This study aimed to investigate the influence of different concentrations of Cu in the TiO_2_ MAO coating on the Ti-30Nb-5Mo alloy. The coatings’ physicochemical properties were evaluated via X-ray diffraction (XRD), X-ray photoelectron spectroscopy (XPS), scanning electron microscopy (SEM), atomic force microscopy (AFM), contact angle, and Vickers microhardness measurements. The microbiology tests were carried out with *Escherichia coli (E. coli)*, *Staphylococcus aureus (S. aureus)*, *Enterococcus faecalis (E. faecalis)*, *Pseudomonas aeruginosa (P. aeruginosa)* bacterial strains, and *Candida albicans (C. albicans)* fungus. The growth and differentiation of adipose tissue-derived mesenchymal stem cells (AMSC) were verified through MTT and alizarin red S.

## 2. Materials and Methods

The Ti-30Nb-5Mo alloy used as substrate was produced by arc-melting with a non-consumable tungsten electrode in a copper crucible. The crucible was cooled with running water inside an argon-controlled atmosphere furnace during the entire melting process. After melting, the ingots were hot-rolled and cut into square samples measuring 10 × 10 × 1 mm^3^. Then, the samples were heat-treated at 1000 °C for 6 h, with a heating and cooling rate of 10 °C/min. More information about this alloy’s cast and properties can be found in the literature [11,12,35].

To carry out the MAO surface modifications, the surfaces of the alloys were sanded with 600 mesh sandpaper to standardize the roughness of the materials. A DC power source, Keysight, N5751A (Keysight, Santa Rosa, CA, USA), was utilized for the MAO process. The oxidation treatment was conducted at room temperature for 1 min, with limited current (2.5 A) and 300 V. The electrolyte solution consisted of 0.35 M calcium acetate monohydrate ((CH_3_COO)_2_Ca·H_2_O), 0.02 M β-glycerol phosphate pentahydrate (C_3_H_7_Na_2_O_6_P·5H_2_O), and 0.1 M magnesium acetate tetrahydrate ((CH_3_COO)_2_Mg·4H_2_O) [36,37,38,39]. To incorporate Cu into the coatings, different concentrations of copper chloride (CuCl_2_) were used (1.5, 2.5, and 3.5 mMol). The samples were labeled as 1.5 Cu, 2.5 Cu, and 3.5 Cu, corresponding to the different CuCl_2_ concentrations.

An optical microscope, Olympus BX51 M (Olympus, Tokyo, Japan), was used to obtain cross-sectional images. In contrast, topography images were acquired using a Zeiss 300 FE scanning electron microscope (SEM) (Carl Zeiss, Oberkochen, Germany). ImageJ software (version 1.53t) was used to analyze micrographs and cross-sectional images, enabling the acquisition of pore size and thickness information.

XRD results were acquired using a MiniFlex600 diffractometer (Rigaku, Tokyo, Japan) with Cu Kα radiation. Data was collected with 10°/min collection time and 0.04° steps.

XPS data were obtained using a SPECS Phoibos 100-MCD5 spectrometer (SPECS Berlin, Germany) with Al Kα radiation. The instrument operated in the fixed analyzer transmission mode at 100 W. Channel width was 1 eV for broad regions, while 0.1 eV was used for detailed regions.

AFM images were acquired using a Park XE-120 microscope (Park, Suwon, Korea) in non-contact mode. 30 × 30 μm^2^ images were taken to estimate each surface roughness.

To determine the wettability of the samples, measurements of the contact angle with deionized water were conducted using the droplet technique on a Krüss DSHAT HTM Reetz GmbH goniometer (HTM Reetz GmbH, Berlin, Germany). Each sample was tested in three different regions.

The Jönsson and Hogmark “law-of-mixtures” model [40,41,42,43] was used to obtain the coatings and substrate hardness values. Leica VMHT equipment (Leica GmbH in Wetzlar, Germany) was used to perform the measurements. A Vickers pyramidal indenter was employed to make indentations on both the substrate and the MAO coatings for 15 s, with loads ranging from 0.98 to 19.6 N. Six indentations were made for each load on the substrates, and 10 were made for each load on the MAO coatings.

Four different bacteria strains (*E. coli*, *S. aureus*, *E. faecalis*, and *P. aeruginosa*) were used to test the growth of microorganisms on the coatings, along with one fungus (*C. albicans*). Before the tests, the samples were autoclaved for 20 min at 121 °C. Then, each sample was placed in tubes with a suspension of a single microorganism in 5 mL of Brain Heart Infusion (BHI, DIFCO, Sparks, MD, USA) medium. The tests were performed in triplicate, and no samples were present in the BHI medium that served as the control. The microorganisms were cultivated at their optimal growth temperature (37 °C for bacteria and 28 °C for fungus) with slow agitation for 24 h. The growth of microorganisms was read via the optical density at a wavelength of 600 nm using a biophotometer (Eppendorf, Hamburg, Germany).

Adipose Mesenchymal Stem Cells (AMSCs) were obtained from the adipose tissue of 3-month-old female lambs of a local slaughterhouse for the cytotoxicity and differentiation tests. The cells were then incubated with the substrates for 24 h, and MTT solution (3-[4,5-dimethylthiazol-2-yl]-2,5-diphenyl-tetrazolium bromide, Sigma-Aldrich, Gillingham, UK) was added to the DMEM medium (Gibco, Loughborough, UK) containing 10% fetal calf serum (FCS, Gibco, Loughborough, UK). Afterwards, the cells were incubated at 37 °C for 3 h with 5% CO_2_. Finally, the culture medium was removed, and the MTT solution was substituted with absolute ethanol (Sigma-Aldrich, Gillingham, UK).

To evaluate the capacity of AMSCs for osteogenic differentiation, the cells were treated with a DMEM medium containing 10% FCS supplemented with 50 μg/mL ascorbic acid, 10 mM ß-glycerophosphate, and 10^7^ M dexamethasone for three weeks. The treated AMSCs were then fixed in 70% ethanol at room temperature for 1 h and washed with distilled water. The cells were then stained with 2% alizarin red S (Carlo Erba, Cornaredo, Italy) for 30 min to detect the presence of Ca deposits, which turned orange-red.

## 3. Results and Discussion

**Figure 1:**
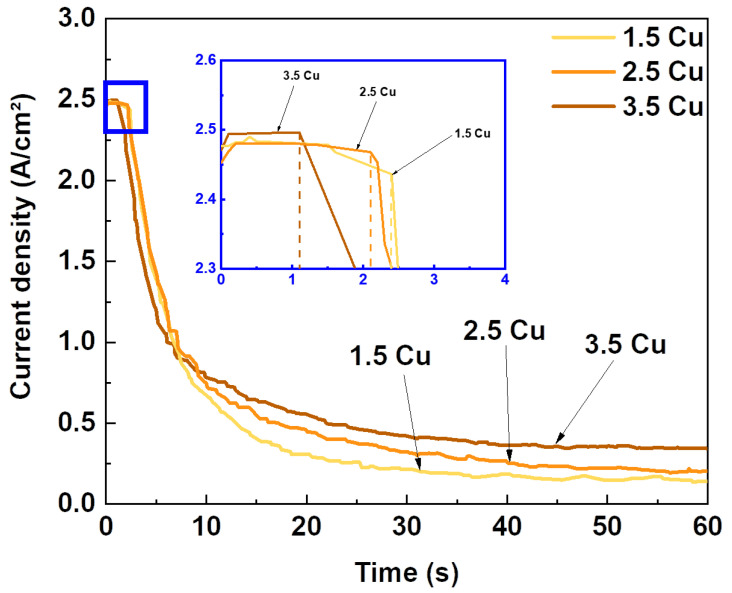
Current density versus time curves during the MAO process with different Cu concentrations.

The circuit’s current was continuously monitored during the MAO treatment process, and Figure 1 displays the current’s evolution over time. The current behavior during the MAO treatment can be divided into galvanostatic and potentiostatic stages [44]. In the galvanostatic stage (highlighted in the blue frame of Figure 1), the current remains constant at the limit value established before the start of the process. During this period, the formation and growth of the compact oxide layer occur [45]. At this stage, the number of plasma discharges is higher due to the more elevated surface conductivity, as the ceramic coating is still growing on the surface [45,46]. The growth of the coating increases the electrical resistance of the sample, causing dielectric barrier breakdown at vulnerable points on the surface, and consequently, the decrease and subsequent stabilization of the current (potentiostatic stage) [39,45].

Figure 1 highlights the instant in which the dielectric breakdown of the circuit occurred using the three different electrolyte solutions, and the corresponding values are presented in Table 1. It was observed that with the increase in the amount of CuCl_2_, the time for the dielectric breakdown decreased. Wang et al. [47] studied the variation of NaPO_3_ concentration during the MAO process and observed that the time for rupture also decreased with increasing electrolyte concentration. According to Wang et al. [47], during the constant voltage of the MAO process, the current versus time curve is directly proportional to the plasma discharge intensity. This discharge intensity is proportional to the energy of the process, thereby accelerating the dielectric breakdown process. Therefore, the increase in CuCl_2_ concentration in this current study led to an increase in the conductivity of the electrolyte solution and, consequently, an increase in the energy of the MAO process.

To verify the increase in process energy, the amount of electrical charge from the treatments of each sample was calculated, and the result is presented in Table 1. Since current (*i*) can be described as the rate at which the electrical charge (*q*) passes through a point or region (Equation (1)) [48], the electrical charge of each process can be calculated by determining the area under each curve shown in Figure 1. Thus, it is observed that the increase in CuCl_2_ in the electrolyte increased the circuit charge.

(1) $$i=\frac{dq}{dt}$$

**Figure 2:**
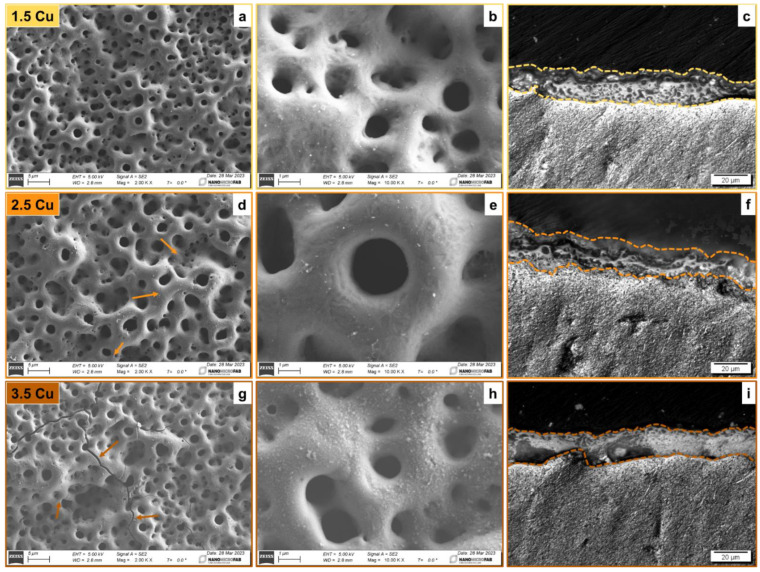
SEM images at 2k× (**a**,**d**,**g**) and 10k× (**b**,**e**,**h**) magnifications, and cross-sectional images (**c**,**f**,**i**) of the 1.5 Cu (**a**–**c**), 2.5 Cu (**d**–**f**), and 3.5 Cu (**g**–**i**) samples.

Figure 2 shows SEM images of the coatings’ topography and optical microscopy images of sample cross-sections after the MAO process. All coatings produced on the Ti-30Nb-5Mo alloy presented a typical morphology of MAO coatings on Ti alloys: rough surface formed by numerous pores in the shape of craters and volcanoes. This typical topography is due to the dielectric breakdown that occurs during the MAO process, which produces bubbles in the contact region between the sample surface and the electrolyte [49]. It was also observed that the increase in electrical charge led to the appearance of cracks on the surface of the coatings. The 1.5 Cu sample shows no cracks, while the 2.5 Cu sample showed some tiny cracks, as indicated in Figure 2d. Finally, in the 3.5 Cu sample, larger cracks can be seen (Figure 2g). Yao et al. [23] studied the incorporation of Cu into Ti alloys. They also observed the appearance of cracks in the coating formed due to the release of stresses generated during the MAO process. The appearance of cracks in the coatings may seem like an adverse effect. However, according to Yao et al. [23], the cracks and the pores can serve as Cu deposition channels in the inner layers of the coating. Thus, the Cu can be preserved longer if the material is applied to implants for extended periods.

Analyzing the SEM images at high magnification (Figure 2b,e,h), the more Cu was used in the MAO process, the more particles deposited on the coatings’ surface were observed. Yao et al. [23] and Zhang et al. [28] also observed nanoparticle accumulation on Cu-doped TiO_2_ coatings. The accumulation of these particles can be caused by increasing the temperature of the MAO process when higher amounts of Cu are added to the electrolyte [50]. 

**Figure 3:**
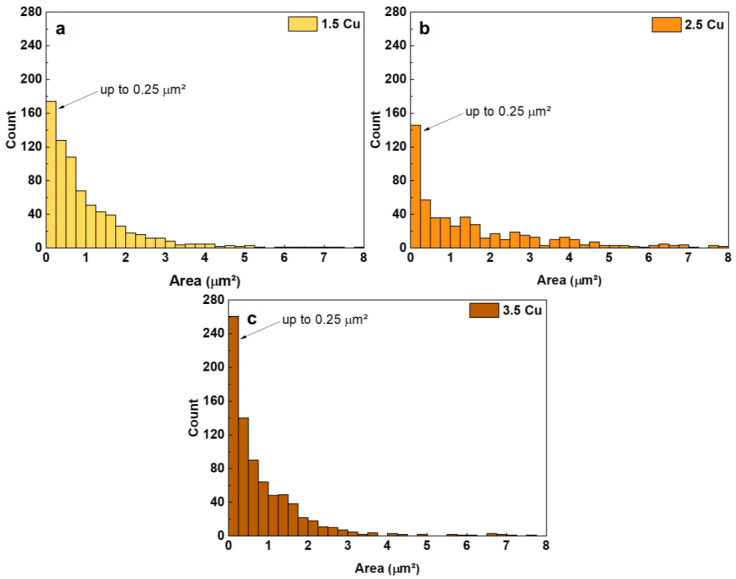
Pore area histograms of the 1.5 Cu (**a**), 2.5 Cu (**b**), and 3.5 Cu (**c**) samples.

**Figure 4:**
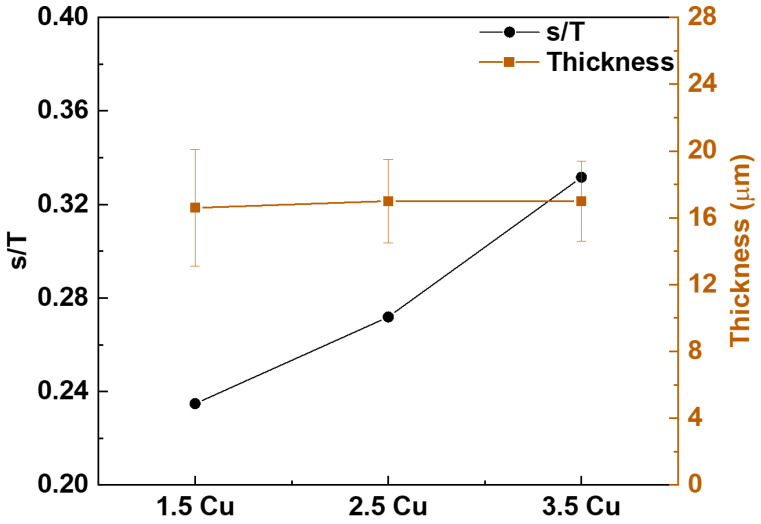
S/T ratio and thickness of the MAO coatings with different Cu concentrations.

The pore size of each surface was calculated using ImageJ software from the SEM images. The results are presented in Figure 3 as a histogram of the pore area. In all coatings, most pores are up to 0.25 μm², with some larger pores up to 8 μm². To have a better estimation of the pore size of each coating, the ratio (s/T) of the number of pores up to 0.25 μm² (s) was determined concerning the total number of pores (T). Thus, the higher the s/T ratio is, the smaller the coating pores are. The result of the s/T ratio is presented in Figure 4 together with the thickness of each coating also determined with ImageJ software. It is observed that the more Cu was used in the electrolyte, the higher the s/T ratio was, i.e., the smaller the pores produced in the coating are.

Regarding the thickness, analysis of the samples’ cross-section showed that the Cu variation was insufficient to modify the coatings’ thickness. The same result was observed by Zhang et al. [51], who studied the variation of Cu in the electrolyte for CP-Ti oxidation and found that the thickness of all coatings was approximately the same size.

**Figure 5:**
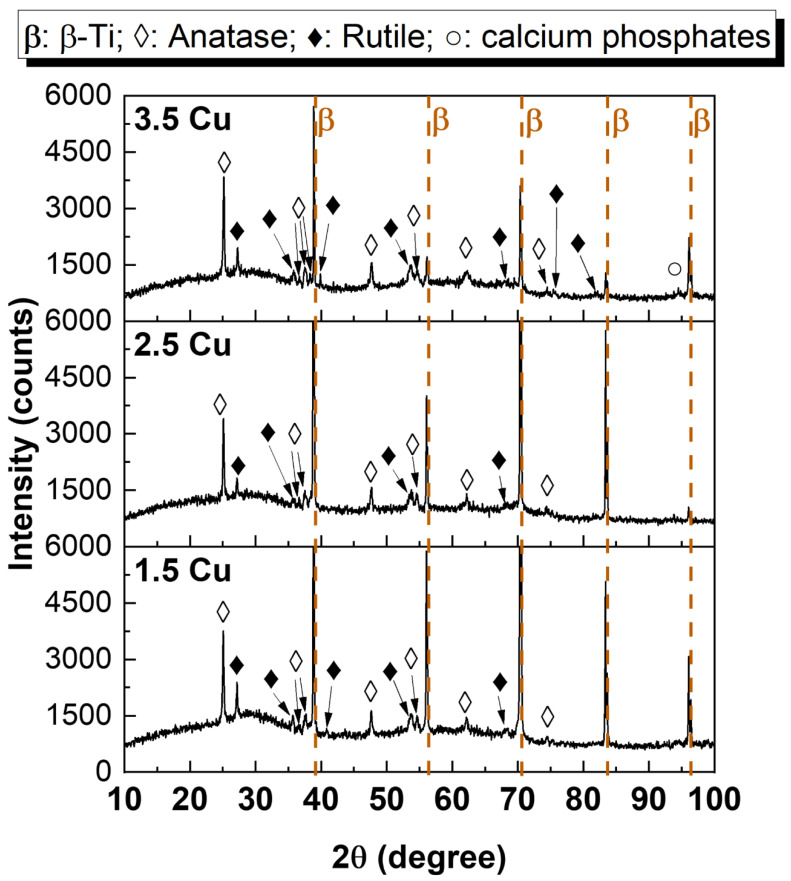
XRD patterns of the MAO coatings with different Cu concentrations.

**Figure 6:**
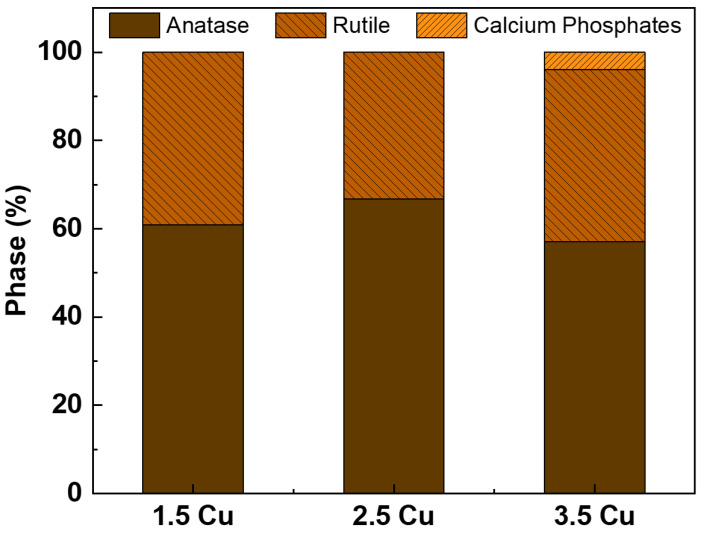
Phase composition by XRD and crystallinity of the MAO coatings with different Cu concentrations.

Figure 5 presents the XRD patterns obtained for each of the MAO coatings. All the coatings only exhibited diffraction peaks corresponding to the anatase and rutile phases of TiO_2_, except for the 3.5 Cu sample, which also showed a calcium phosphate peak. Due to the low Cu concentration, no Cu-containing phases were detected [28]. Using Equation (2) [52], it was possible to calculate the proportion of each phase, and the results are presented in Figure 6. The intensities of each peak in the diffractogram were considered to calculate the percentage of each phase. The results showed no significant variations between the percentages of anatase and rutile phases of TiO_2_. Several other studies that also incorporated Cu into MAO coatings on Ti varying Cu concentrations did not report any changes in the XRD phase composition [23,51,53,54].

Standards were taken from the American Mineralogist Crystal Structure Database: β-Ti (0013128), anatase (0019093), rutile (0001737), and calcium phosphate (0020245).

(2) $$\%phase=\frac{\sum I_{phase}}{\sum I_{all\;phase}}$$

**Figure 7:**
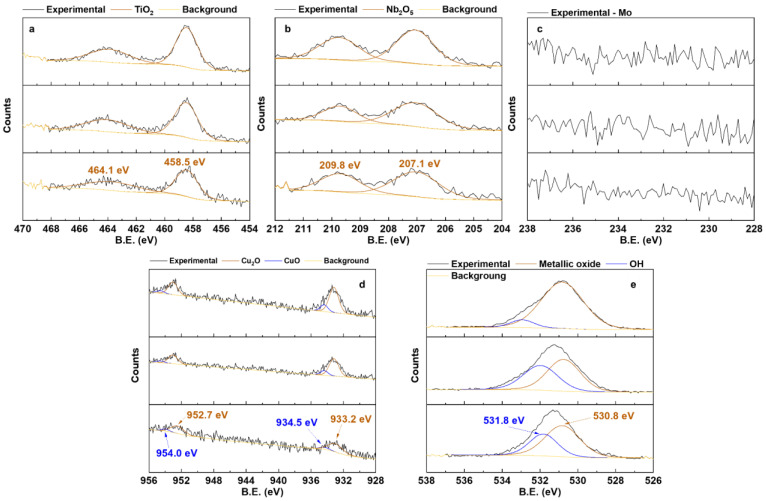
High-resolution XPS of the Ti (**a**), Nb (**b**), Mo (**c**), Cu (**d**), and O (**e**) present in the MAO coatings.

Figure 7 shows the high-resolution XPS spectra of the alloying elements incorporated into the coating. Regarding the alloying elements, doublets related to TiO_2_ (Figure 7a) and Nb_2_O_5_ (Figure 7b) were observed at 458.5 and 464.1 eV [38,44,55,56,57] and 207.1 e 209.8 eV [44], respectively. Mo is present in low quantities in the alloy (5% by weight) and is also known as a valve metal. Because of this, the oxidation of Mo through the MAO method is less favorable from an energetic point of view [58,59]. Consequently, no peaks of this element were detected (Figure 7c). Regarding the Cu incorporated into the coating by the MAO process, doublets of Cu_2_O at 933.2 and 952.0 eV, and CuO at 935.7 and 955.8 eV were detected (Figure 7d) [54,60,61]. Lastly, in the O1s spectrum (Figure 7e), peaks of metallic oxides at 530.8 eV and hydroxide (OH) at 531.8 eV [56,62] were registered. Analyzing the composition of each Cu oxide, it was observed that there was an increase in the CuO/Cu_2_O ratio (0.15, 0.21, and 0.37) as the Cu concentration in the electrolyte increased. Yang et al. [61] reported the same effect when increasing Cu incorporation in CP-Ti samples. The following redox reactions occur during the MAO process to form CuO and Cu_2_O [60]. Thus, with more CuCl_2_ available in the electrolyte, the redux reaction of 2Cu_2_O into 4CuO occurs, increasing the CuO/Cu_2_O ratio.

$$\begin{array}{c} {\mathrm{CuCl}}_{2}+2H_{2}O\to \mathrm{Cu}{\left(\mathrm{OH}\right)}_{2}+2\mathrm{HCl}\\ 2\mathrm{Cu}{\left(\mathrm{OH}\right)}_{2}\to {\mathrm{Cu}}_{2}O+H_{2}O\\ 2{\mathrm{Cu}}_{2}O+O_{2}\to 4\mathrm{CuO} \end{array}$$

**Figure 8:**
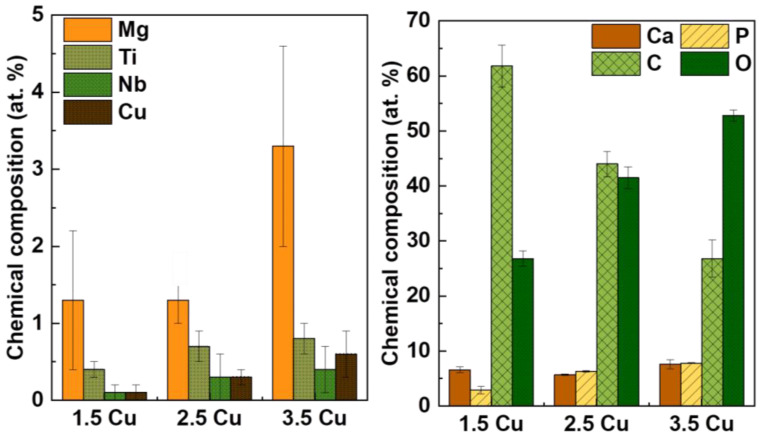
Chemical composition obtained by XPS of the MAO coatings.

Figure 8 shows the composition of the elements identified by XPS. It was observed that with an increase in Cu concentration in the electrolyte, the concentration of all elements, except for C and Ca, grew due to the increase in electrolyte conductivity. Kuroda et al. [63] explained that the energy of the process influences the flow of electrons through the coating produced by the MAO method, so higher electrical conductivity increases the rate of ions reaching the coating. 

**Figure 9:**
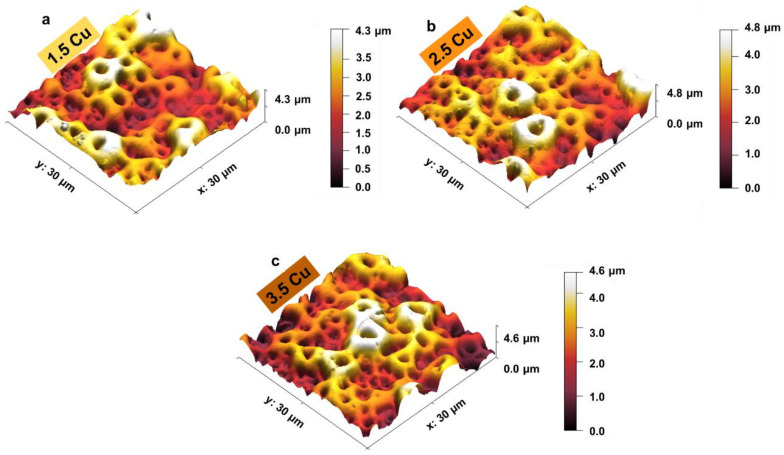
Three-dimensional topography images of the 1.5 Cu (**a**), 2.5 Cu (**b**), and 3.5 Cu (**c**) samples.

**Figure 10:**
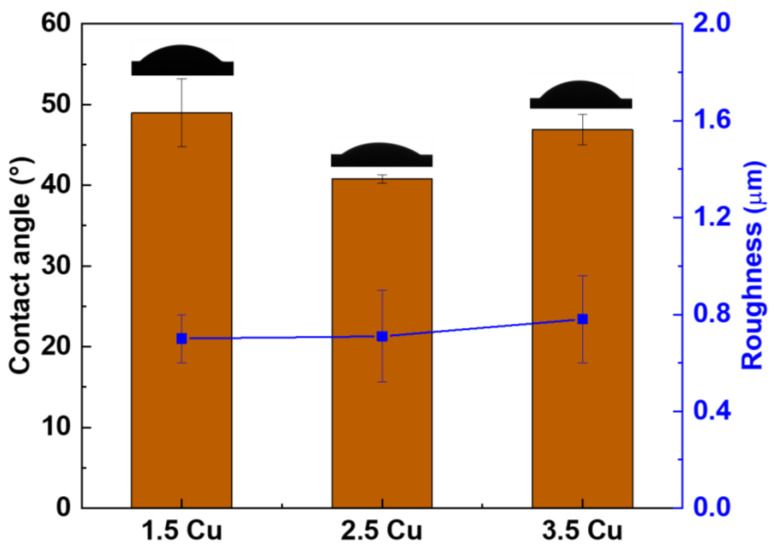
Contact angle and surface roughness of the MAO coatings with different Cu concentrations.

The 3D topographic images of the coating surfaces obtained by the AFM technique are presented in Figure 9. From these images, it was possible to determine the average surface roughness (RMS), and the results are presented in Figure 10 (blue line), along with the contact angle values with distilled water (bars). It was observed that the variation of CuCl_2_ in the electrolyte did not significantly change the coatings’ roughness and contact angle values. Wang et al. [54], who studied the effect of Cu on the production of MAO coatings on CP-Ti, also obtained similar results: there were no significant changes in the roughness and wettability of the surfaces. 

Although all coatings produced on the Ti-30Nb-5Mo alloy substrates show no considerable variations in contact angle, their values are below 90°, indicating the hydrophilicity of the coatings. This situation is because TiO_2_, the main component of the coatings, has a high surface polarity, making it more susceptible to bonding with water molecules [39,62,64].

**Figure 11:**
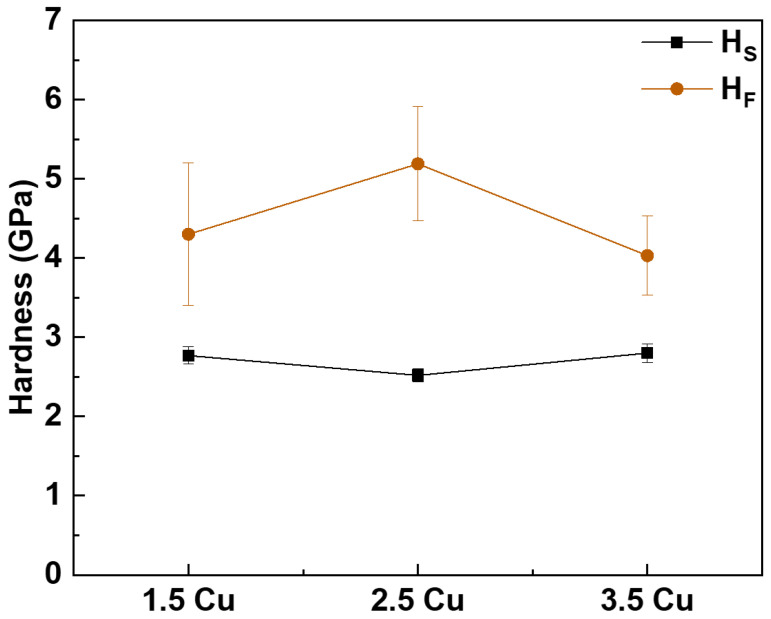
Hardness of the substrates (H_s_) and the MAO coatings (H_f_) with different Cu concentrations.

The hardness of the substrate and coatings after each MAO process with different electrolyte solutions are shown in Figure 11. The different electrolytes did not considerably change the substrate’s hardness, which varied between 2.5 and 2.8 GPa. The values obtained are similar to those reported by Cardoso et al. [11] (2.8 ± 0.1 GPa), who studied the mechanical properties of the Ti-30Nb-5Mo alloy.

It was not possible to observe any trend in the variation of coating hardness with increasing Cu content. The hardness of the 1.5 Cu coating was 4.3 ± 0.9 GPa. This value increased to 5.2 ± 0.7 GPa for the 2.5 Cu sample and decreased again to 4.0 ± 0.5 GPa for the sample with the highest Cu concentration (3.5 Cu). The slight variation in hardness values can be explained by the low variation in Cu concentration between the different coatings. However, all values obtained are not very different from the values found in the literature for porous TiO_2_ MAO coatings on Ti alloys (1.4 GPa [41], 2.2 GPa [65], 2.9 GPa [66], and 3.9 GPa [33]). The coatings’ higher hardness values are important because they provide greater strength to the material and protect the substrate from wear [67,68].

**Figure 12:**
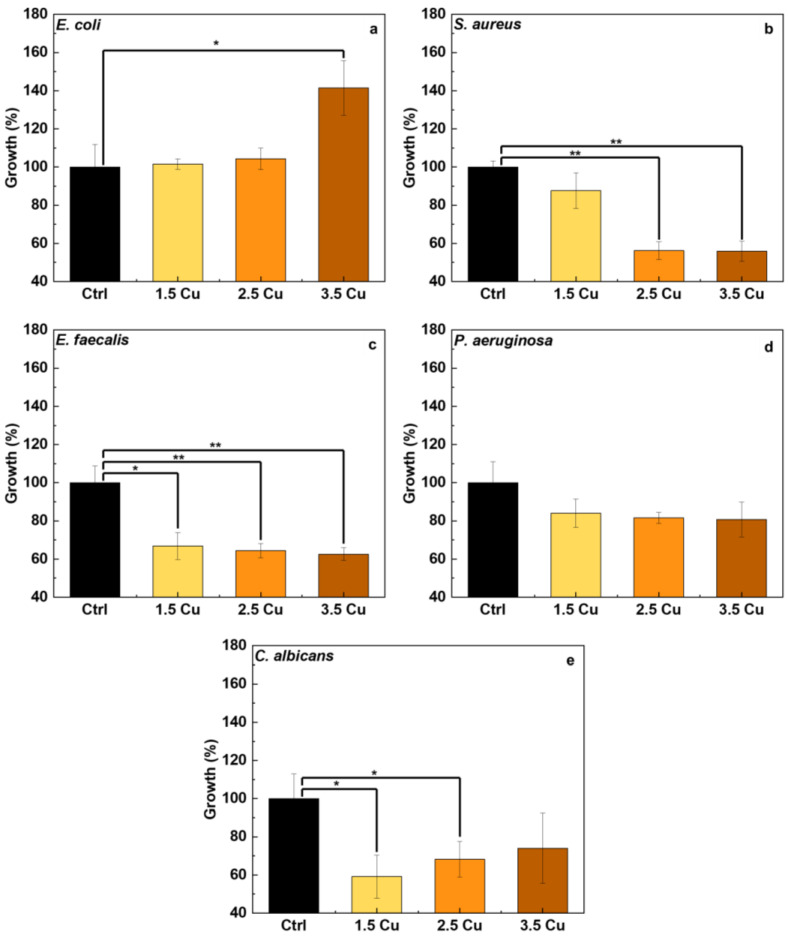
*E. coli* (**a**), *S. aureus* (**b**), *E. faecalis* (**c**), *P. aeruginosa* (**d**), and *C. albicans* (**e**) growth on the MAO coatings with different Cu concentrations. * *p* < 0.05, ** *p* < 0.01, compared with the control group.

Figure 12 shows the growth of microorganisms on the surface of different coatings incorporated with Cu. Among all the microorganisms tested, only the *E. coli* bacteria maintained a growth rate equal to or higher than that of the control. In other words, incorporating Cu did not affect the growth of this bacteria strain. Moreover, sample 3.5 Cu showed a higher growth of *E. coli* than the control sample. This may have occurred due to the increase in the number of cracks in the coating, which provided a more favorable environment for bacteria with lower concentrations of Cu.

On the other hand, the incorporation of Cu was effective in significantly reducing the growth of the bacteria strains, such as *S. aureus* (by 44%), *E. faecalis* (by 37%), and *P. aeruginosa* (by 19%), as well as the fungus *C. albicans* (by 41%). As shown in the chemical composition results obtained by XPS (Figure 8), the concentration of Cu incorporated into the coatings did not vary significantly. This may explain why Cu concentration has no apparent influence on the results obtained for *E. faecalis* and *P. aeruginosa*.

There is no consensus in the literature on the mechanisms of the bactericidal effect of Cu. Some authors attribute the effect to the absorption of Cu ions by bacteria, which leads to the formation of cavities in the bacterial cell wall [69]. In contrast, other authors suggest that there must be a significant interaction between the Cu nanoparticle and the microorganism for contact death to occur [70]. Yao et al. [23] believe that both mechanisms (contact and release of ions) are responsible for the bactericidal effect of Cu. Shimabukuro et al. [71] studied the incorporation of Cu into CP-Ti and analyzed the bactericidal effect of the coating against *E. coli* and *S. aureus*. According to the authors [71], the effectiveness of Cu is more prominent against *S. aureus* than *E. coli*, requiring a higher minimum inhibitory concentration (MIC) of Cu to inhibit *E. coli* growth. Furthermore, the authors [71] found that the bacterial property of the coating is activated by contact with Cu, rather than through the release of ions. This situation helps to explain why there was increased growth of *E. coli* in the 3.5 Cu sample, as more bacteria colonies can form inside the cracks where there is less penetration of Cu. Furthermore, Cu_2_O exhibits higher bactericidal efficiency than CuO [60], and, as shown in the XPS results of this present study, the CuO/Cu_2_O ratio increased, i.e., there was more CuO on the coating surface.

**Figure 13:**
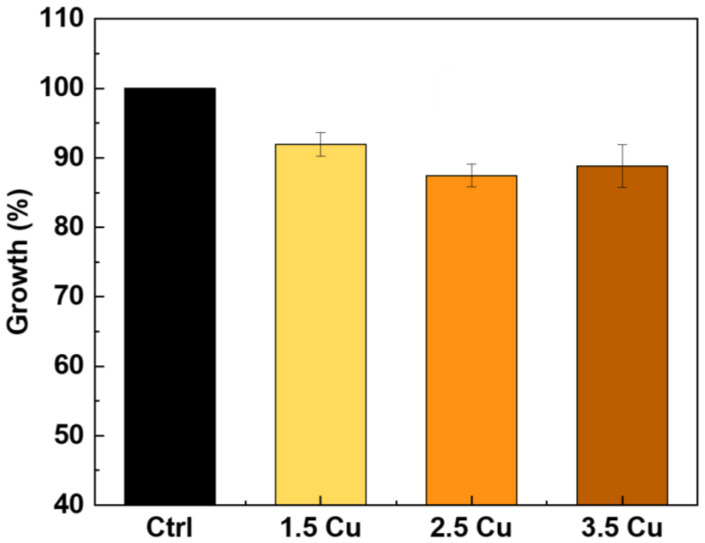
AMSCs growth on the MAO coatings with different Cu concentrations.

The growth of AMSC cells, obtained via the MTT method, is shown in Figure 13. According to the ISO 10993-5 standard [72], up to 70% of cell viability is required for non-cytotoxicity [73,74,75]. Furthermore, the standard establishes that 100% cell viability samples are not cytotoxic (grade 0). Samples between 80% and 100% viability are slightly cytotoxic (grade 1). Samples with viability between 50% and 80% are mildly cytotoxic (grade 2), while samples between 30% and 50% are moderately cytotoxic (grade 3). Samples with viability below 30% are severely cytotoxic (grade 4) [72,76]. In this presented study, cell growth on all coatings was approximately 90%. Therefore, they fall into grade 1 of cytotoxicity.

**Figure 14:**
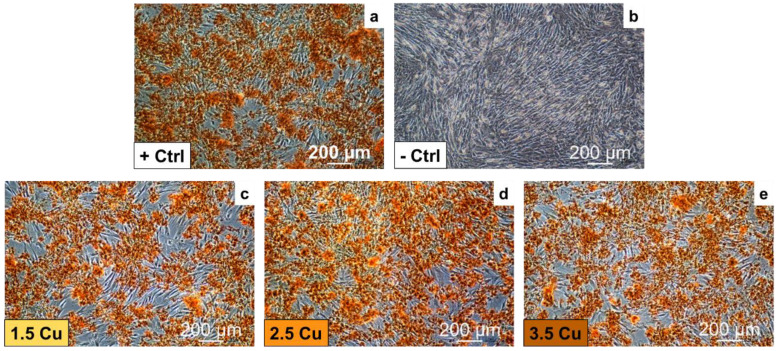
Alizarin red S staining of AMSCs differentiated in vitro into the osteogenic lineage on positive control (**a**), negative control (**b**), and on 1.5 Cu (**c**), 2.5 Cu (**d**), and 3.5 Cu (**e**) coating samples.

In Figure 14, the cell monolayer images of the samples with different concentrations of Cu, stained with alizarin red S to highlight in red the Ca deposits, are presented. The positive control (+Ctrl) is the AMSCs that have differentiated into the osteogenic lineage without the substrate (Figure 14a), while the negative control (-Ctrl) is represented by non-differentiated AMSCs (Figure 14b). All the samples’ images resemble the positive control. Therefore, the Cu levels used did not alter the AMSCs’ differentiation into the osteogenic lineage. Zhu et al. [53] and Zhang et al. [60] studied the incorporation of Cu into TiO_2_ coatings produced by the MAO method. They concluded that the coatings promoted the differentiation of MG63 [53] and MC3T3-E1 [60] cells. This phenomenon occurred because the coatings are porous and provide a higher contact area with the medium used. This, in turn, can facilitate the adsorption of proteins that promote cell differentiation [60]. Furthermore, it has been reported that the expression of insulin-like growth factor—1 (IGF-1, which plays an essential role in the growth and development of osteoblasts) is increased by Cu [53].

## 4. Conclusions

With the results obtained in this study, it was possible to verify how the increase in Cu ions (from 1.5 to 3.5 mMol) in the electrolyte used in the MAO process of the Ti-30Nb-5Mo alloy influenced the morphology and properties of the coatings. The XRD patterns showed that the crystalline part of the coatings comprises TiO_2_ (anatase and rutile phases). A small amount of calcium phosphate (~4%) was detected in the sample with the highest Cu concentration. It was found via XPS that the CuO/Cu_2_O ratio in the coatings increased from 0.15 to 0.37 due to Cu incorporation. The coating samples showed no significant variations in roughness (~0.7 to 0.8 μm) and wettability (~40 to 50°). All the coatings were hydrophilic (<90°). The hardness of the coatings also did not show significant variations, the values remaining higher (~4 to 5 GPa) compared to the substrate hardness (2.8 GPa). The incorporation of Cu into the coatings improved the antimicrobial activity of the surface since the growth of *S. aureus*, *E. faecalis*, *P. aeruginosa*, and *C. albicans* was approximately 56%, 63%, 81%, and 59% (concerning 100% of control), respectively. However, the growth of *E. coli* was not affected by the levels of Cu incorporated into the coatings. The AMSC cell growth on all the surfaces was above 90%, proving the non-cytotoxicity of the prepared coatings. Moreover, adding Cu (up to 3.5 mMol) did not affect the differentiation of AMSCs into the osteogenic lineage.

## Figures and Tables

**Table 1:** Dielectric breakdown time and electric charge density during the MAO process with different Cu concentrations.

Sample	Dielectric Breakdown (s)	Electric Charge Density of the Entire Process (C/cm²)
1.5 Cu	2.4	27.3
2.5 Cu	2.1	32.6
3.5 Cu	1.1	36.7

## Data Availability

The obtained data are available upon a reasonable request to the corresponding author.
